# Implementation and evaluation of amyloidosis subtyping by laser-capture microdissection and tandem mass spectrometry

**DOI:** 10.1186/s12014-016-9133-x

**Published:** 2016-10-27

**Authors:** Peter Mollee, Samuel Boros, Dorothy Loo, Jayde E. Ruelcke, Vanessa A. Lakis, Kim-Anh Lê Cao, Patricia Renaut, Michelle M. Hill

**Affiliations:** 1 Amyloidosis Centre, Princess Alexandra Hospital, Brisbane, QLD 4102 Australia; 2 Anatomical Pathology Department, Pathology Queensland, Princess Alexandra Hospital, Brisbane, QLD Australia; 3 The University of Queensland Diamantina Institute, The University of Queensland, Level 5, Translational Research Institute, 37 Kent Street, Woolloongabba, QLD 4102 Australia

**Keywords:** Amyloid, Mass spectrometry, Laser capture microdissection, Diagnosis, Proteomics

## Abstract

**Background:**

Correct identification of the amyloidosis-causing protein is crucial for clinical management. Recently the Mayo Clinic reported laser-capture microdissection (LCM) with liquid chromatography-coupled tandem mass spectrometry (MS/MS) as a new diagnostic tool for amyloid diagnosis. Here, we report an independent implementation of this proteomic diagnostics method at the Princess Alexandra Hospital Amyloidosis Centre in Brisbane, Australia.

**Results:**

From 2010 to 2014, 138 biopsies received from 35 different organ sites were analysed by LCM-MS/MS using Congo Red staining to visualise amyloid deposits. There was insufficient tissue in the block for LCM for 7 cases. An amyloid forming protein was ultimately identified in 121 out of 131 attempted cases (94 %). Of the 121 successful cases, the Mayo Clinic amyloid proteomic signature (at least two of Serum Amyloid P, ApoE and ApoA4) was detected in 92 (76 %). Low levels of additional amyloid forming proteins were frequently identified with the main amyloid forming protein, which may reflect co-deposition of fibrils. Furthermore, vitronectin and clusterin were frequently identified in our samples. Adding vitronectin to the amyloid signature increases the number of positive cases, suggesting a potential 4th protein for the signature. In terms of clinical impact, amyloid typing by immunohistochemistry was attempted in 88 cases, reported as diagnostic in 39, however, 5 were subsequently revealed by proteomic analysis to be incorrect. Overall, the referring clinician’s diagnosis of amyloid subtype was altered by proteomic analysis in 24 % of cases. While LCM-MS/MS was highly robust in protein identification, clinical information was still required for subtyping, particularly for systemic versus localized amyloidosis.

**Conclusions:**

This study reports the independent implementation and evaluation of a proteomics-based diagnostic for amyloidosis subtyping. Our results support LCM-MS/MS as a powerful new diagnostic technique for amyloidosis, but also identified some challenges and further development opportunities.

**Electronic supplementary material:**

The online version of this article (doi:10.1186/s12014-016-9133-x) contains supplementary material, which is available to authorized users.

## Background

Amyloidosis is a rare but devastating condition caused by deposition of misfolded proteins as aggregates in the extracellular tissues of the body, leading to impairment of organ function [1]. Many, but not a limitless number of proteins can cause amyloidosis [2]. The most common are immunoglobulin light chain (AL), transthyretin (ATTR), serum amyloid A protein (AA) and the alpha chain of fibrinogen (AFib). Treatment and prognosis depend on identifying the culprit protein [3]. Treatment aimed at reducing the amyloidogenic protein involves preventing production and aggregation of these misfolded proteins. For example, in AL amyloidosis chemotherapy is required to kill the clonal bone marrow plasma cells that produce the pathologic immunoglobulin light chain. Such therapies are inappropriate and in fact harmful for other types of amyloidosis. Similarly, accurate diagnosis of amyloid subtype is critical to guide organ transplantation to replace the organ that manufactures the pathogenic protein in hereditary amyloidosis [4]. Correct identification of the causal amyloid protein is thus absolutely crucial for clinical management in order to avoid misdiagnosis and inappropriate, potentially harmful treatment, to assess prognosis, and to offer genetic counselling if relevant.

In clinical diagnostic laboratories the diagnosis of amyloidosis is made by histological examination of tissue biopsy samples with the presence of amyloidosis demonstrated by the Congo red immunohistochemical stain which results in a pale ‘salmon-pink’ staining that shows typical birefringence and dichroism effects when examined under polarised light microscopy [5]. Subtyping of the amyloid deposits is made by the use of immunohistochemical (or immunofluorescence) stains for the various potential amyloid proteins [6]. This is problematic as, outside of highly specialised centres, these stains are often unreliable giving both false positive and false negative findings [7, 8]. Recently, the Mayo Clinic has established the use of laser capture microdissection (LCM) followed by tandem mass spectrometry (MS) to identify the subtype of amyloid with a high degree of confidence from clinical biopsy samples [9–11]. In this study we report the implementation and evaluation of this novel diagnostic technique at a tertiary referral hospital in Brisbane Australia over 5 years.

## Methods

### Clinical specimens

The study was approved by the Princess Alexandra Hospital Ethics Committee. Diagnostic formalin fixed paraffin embedded tissue biopsy samples from 138 patients referred to the Princess Alexandra Hospital Amyloidosis Centre between 2010 and 2014 were used for this study. Seven of these had insufficient tissue remaining in the block for LCM. Clinicians referring patient biopsy samples were required to complete a baseline proforma of clinical details of the case and were asked to indicate their clinical diagnosis of amyloid subtype.

### Specimen microdissection and processing

Ten micron sections were cut from formalin fixed paraffin embedded tissue onto Arcturus PEN membrane glass slides. Tissue sections were deparaffinised and stained with Congo red. The stained sections were rinsed and thoroughly air-dried. Congo red positive areas were dissected using an Arcturus LCM system. Amyloid regions were processed according to the Stratagene FFPE protein extraction protocol. Briefly, LCM samples were incubated with FFPE protein extraction solution at 90 °C for 10 min, followed by 60 °C for 120 min and alkylation with 167 mM of iodoacetamide for 30 min. Samples were diluted 10 times with 50 mM ammonium bicarbonate and 10 % acetonitrile for overnight digestion with 0.1 μg/μl trypsin at 37 °C. Trypsin inactivation was achieved by acidifying samples with 0.1 % formic acid. Buffer volumes were adjusted on the basis of the LCM size, however, all concentrations remained constant.

### Tandem mass spectrometry and classification

Extracted peptides (8 μl) were analysed with high performance liquid chromatography (HPLC) coupled Chip-cube QTOF mass spectrometer 6520 or 6530 (Agilent Technologies). Solvent A composition was 0.1 % formic acid, solvent B was 0.1 % formic acid, 90 % acetonitrile. Samples were desalted on the enrichment column of G4240-62010 C18 HPLC chip for 12 s prior to a 20 min gradient from 5 to 50 % B. HPLC loading pump was set to 2.5 % B, flow rate of 3 μl/min while analytical pump was set to 5 % B and flow rate of 0.3 μl/min. Mass spectrometer was programmed to acquire 8 MS and 4 MS/MS spectra/sec with dynamic exclusion after 2 MS/MS and released after 0.2 min.

Mass spectrometry data was analysed using Spectrum Mill search engine against NCBInr human database with carbamidomethylation cysteine as fixed modification, and oxidized methionine, pyroglutamic acid N-term, and deamidated asparagine as variable modifications. Protein identification cut-offs were protein score >11, peptide score >10 and % scored peak intensity >60. For each case the protein score for known amyloidogeneic proteins and known amyloid-associated proteins was calculated. Amyloid subtype was ascribed to the amyloidogeneic protein with the highest score.

Distinction between localized and systemic light chain amyloidosis was based on clinical information. Localized amyloidosis had amyloid deposits in only one site in the absence of a circulating clonal light chain. Systemic AL amyloidosis had an identified clonal plasma cell or lymphoproliferative population distant from the amyloid site.

### Statistical analysis

All statistical analyses were performed using the R statistical software (https://www.R-project.org/). Shapiro–Wilk tests confirmed the non normality of the data therefore Spearman’s rank correlation were used to assess correlation between parameters and non parametric Mann–Whitney U test were used to assess median differences in those parameters between negative and positive Mayo signature. Fisher’s exact tests were performed to evaluate the significance of the relative proportion of samples found within proteins of interest.

## Results

### LCM-MS/MS identification of amyloid forming proteins

**Fig. 1:**
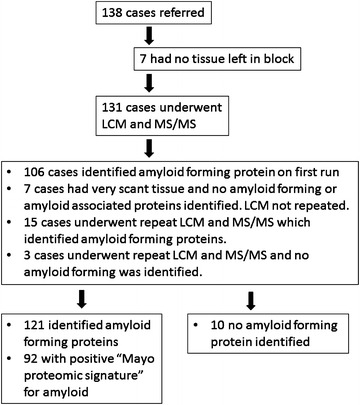
Flowchart for samples analysed

LCM-MS/MS was attempted on 131 clinical biopsy samples referred to the Princess Alexandra Hospital Amyloidosis Centre between April 2010 and December 2014 (Fig. 1). Seven additional cases were not analysed due to lack of tissue in the block. The biopsy samples came from 35 different organ sites, with the most common organs of origin being heart (n = 24), kidney (n = 17), colon (n = 8) and small bowel (n = 7). Of the 131 cases an amyloid forming protein was identified in 106 cases in the first run. Seven cases where no amyloid protein was identified were due to very scant amyloid deposits in the dissected tissue and further LCM was not attempted. Repeat LCM was performed for 18 cases of which 15 had amyloid forming proteins identified. Ultimately, an amyloid forming protein was identified in 121 of 131 cases, and subtyped based on the amyloid forming protein of highest score (Table 1; Additional file 1: Table S1).

**Table 1:** Major amyloid fibril type identified by LCM-MS/MS

Amyloidosis subtype	AL-lambda	AL-kappa	AH	Localised lambda	Localised kappa	ATTRwt	ATTRmut	AA
Amyloid protein/gene	Immunoglobulin lamda light chain	Immunoglobulin kappa light chain	Immunoglobulin heavy chain	Immunoglobulin lamda light chain	Immunoglobulin kappa light chain	Transthyretin	Transthyretin	Serum amyloid A (SAA)
Number of subtype cases	32	13	5	17	9	25	6	7
Amyloid forming proteins								
Lambda light chain	32			17	1	2		
Kappa light chain	4	13	2	5	9	4		1
Ig heavy chain	8		5	6	6	4	1	3
Transthyretin		1		3	3	25	6	
Apolipoprotein A1	2	3		7	3			
Serum amyloid A (SAA)								7
Fibrinogen alpha chain	2	1		2	1			
Lysozyme	1	1		1				
Cases with low levels of 2nd amyloid forming protein	34 %	46 %	40 %	81 %	89 %	20 %	17 %	57 %
Amyloid associated proteins								
ApoE	21	12	2	16	7	19	6	5
SAP	16	8		13	6	24	6	4
ApoA4	23	10	1	16	8	22	6	2
Amyloid signature	63 %	77 %	0 %	100 %	78 %	92 %	100 %	57 %

Not shown in the table: 1 case of TGFb, 2 cases of semenogelin, 2 cases of AFib, 2 cases of ALect

A lower level of additional amyloid forming protein(s) was also identified in a number of cases. Notably, secondary amyloid forming proteins were identified in 23 out of 26 localized AL cases (88.46 %). In comparison, secondary amyloid forming proteins were identified in 18 out of 46 systemic AL cases (40 %). The proportion of cases with secondary amyloid forming proteins was determined to be significantly different between localised and systemic AL diagnoses (p value = 5.068e−05, Fisher’s exact test).

Various other proteins were also identified by LCM-MS/MS of amyloid extracts. Of particular interest is the presence of proteins typically known to be co-located in amyloid deposits which helps confirm that the microdissected tissue is amyloid. Typical amyloid-associated proteins were identified in the following number of cases: vitronectin (n = 104), apolipoprotein A4 (ApoA4, n = 90), apolipoprotein E (ApoE, n = 94), serum amyloid P component (SAP, n = 83), and clusterin (n = 45). ApoA4, ApoE and SAP were previously suggested by the Mayo Clinic as a proteomics signature for amyloid, with any two out of the three proteins being sufficient to confirm amyloid [12]. Of the 121 successfully diagnosed cases, 92 cases (76 %) were positive for the Mayo amyloid signature, with distribution shown in Table 2. Mann–Whitney U test was used to determine if presence of a Mayo amyloid signature (e.g. positive or negative) was associated with a more confident amyloid diagnosis, as indicated by higher Amyloid score and Amyloid % coverage. The p values obtained (p = 1.838e−05 and 0.0006725, for Amyloid score and Amyloid % coverage respectively) indicate that the samples with a positive Mayo signature provided more confident amyloid diagnoses.

**Table 2:** Number of cases identified with proteins from the Mayo amyloid signature

	ApoE	SAP	ApoA4	2 out of 3
Negative	27	38	31	29
Positive	94	83	90	92

In addition to ApoE, SAP and ApoA4, our dataset showed frequent identification of vitronectin and clusterin in amyloid deposit. Therefore, we investigated the value of adding either protein to the Mayo amyloid signature. Clusterin was detected in 42 out of 92 cases positive for the Mayo amyloid signature, but only 3 out of 29 cases negative for the Mayo amyloid signature. It was deemed of limited additional utility. On the other hand, vitronectin was detected in 88 out of 92 samples which were positive for the Mayo amyloid signature, and 16 out of 29 samples negative for Mayo amyloid signature. A fisher exact test was performed to evaluate whether the relative proportion of samples with a presence of vitronectin was independent of the number of samples that were found to have a positive or negative Mayo signature. A significant association was found between the number of samples with the presence of vitronectin and the Mayo signature (p value = 9.037e−07, Fisher’s exact test). When we add vitronectin into the mix and evaluated how many positive diagnoses are identified if two of four proteins (i.e. ApoE, ApoA4, SAP and Vitronectin) are identified the total number of positive diagnoses increases from a Mayo positive signature in 92 cases to a positive amyloid signature in 104 cases.

### Impact of LCM-MS/MS on immunohistochemistical and clinical diagnosis of amyloid subtype

Amyloid typing by immunohistochemistry (IHC) had been attempted in 88 cases, with 49 reported as non-diagnostic or uninterpretable, and 39 as diagnostic (Additional file 1: Table 1). Five of the latter were subsequently revealed by proteomic analysis to be incorrect. Three of the cases with a LCM-MS/MS diagnosis of ATTR were elderly men with isolated cardiac amyloidosis, no proteinuria and no paraprotein or clonal light chain. These three were misdiagnosed on IHC as AL (n = 2) and AA (n = 1). One case with a LCM-MS/MS diagnosis of AL was diagnosed on IHC as AA amyloidosis. This case had biopsy proven renal involvement, cardiac involvement, peripheral neuropathy and IgA lambda paraprotein. The final case was diagnosed by IHC as AA but by LCM-MS/MS was diagnosed as AL amyloidosis. This case presented with hepatic, renal, cardiac and gastrointestinal organ involvement and had an IgG lambda paraprotein.

Overall, the referring clinician’s diagnosis of amyloid subtype was altered by proteomic analysis in 24 % of cases. Most commonly (n = 15) the referring clinician indicated they were uncertain as to the amyloid subtype, but in 11 cases the clinical diagnosis was AL amyloidosis but LCM-LC-MS/MS demonstrated the actual subtype was ATTR (n = 7), AA (n = 2) and AH (n = 2).

## Discussion

Here we report our results with LCM-MS/MS for amyloidosis subtyping in a referral centre with a relatively small case load. Since establishing the procedures within the proteomics facility of a research institution (The University of Queensland Diamantina Institute) associated with the hospital in 2010, we have successfully analysed samples in batches. Our instrumentation and analysis software was different to that reported by the Mayo Clinic [9–11, 13, 14]. The overall success rate for LCM-MS/MS amyloid identification was 121 out of 138 referred cases (88 %), or 121 out of 131 attempted cases (92 %). In contrast, our IHC success rate for a subset of the sample was 45 % (39 out of 87) and 5 of these were incorrect. These results highlight the problem with immunohistochemistry for subtyping outside of centres of expertise, but also demonstrate the robustness of LCM-MS/MS method.

While the success rate for LCM-MS/MS amyloid identification was high, interpretation is not always straightforward and we note several caveats with this technique. Firstly, more than one amyloid forming proteins are identified in many samples. In these cases, we classified by the most abundant protein as per Vrana et al. [9]. However, in some cases the top two proteins were very similar in total intensity. In rare occasions SAA protein has been noted as a contaminant in samples analysed after SAA amyloid samples. In general amyloid proteins are highly hydrophobic proteins which require intensive conditioning procedures to elute off reverse phase columns. To prevent leaching of contaminants to subsequent analysis, larger LCM samples and samples with SAA protein should be diluted, or queued at the end of a batch. Secondly, in cases of AL amyloidosis, clonality of identified immunoglobulins is strengthened by demonstrating the light chain or heavy chain variable region rather than the constant region, which is not always the case. A new bioinformatic workflow was recently reported to detect light chain variable peptides and may be a useful tool for determining clonality [15]. Furthermore, clinical information is still required for subtyping. For example, systemic versus localized AL amyloidosis is classified by clinical information. Hence, a system to integrate clinical and MS/MS data is required. Finally, repeat LCM is required for some samples, adding time and cost to the diagnosis. Triaging of small LCM samples could be implemented to reduce time and resource wastage. Alternatively, imaging mass spectrometry technique has recently been reported for localized detection of amyloid peptides in situ, and could be further evaluated [16].

There are several limitations in the current study, which should be addressed in the future. Firstly, not all of the cases were fully worked up with all immunohistochemical staining and so a “gold standard” diagnosis could not always be assigned. In some cases the initial MS failed for various reasons and a second LCM and MS analysis was required to attain a diagnosis. Secondly, while our data suggest vitronectin to be a potential new amyloid signature protein, LCM-MS/MS demonstrating the absence of vitronectin in adjacent normal tissue and in tissues involved by non-amyloid pathology would be required. Finally, there were few hereditary amyloid cases in our cohort, and our methodology is not optimized for mutation detection.

## Conclusions

In conclusion, our study supports LCM-MS/MS as a robust diagnostic platform for identification of fibril composition of amyloid deposits in clinical biopsy samples. Further optimization and standardization of the methodology will be required to add LCM-MS/MS as an additional arsenal in amyloid subtyping. Future work should standardize the sample preparation methodology, establish a specialized database to account for immunoglobulins and amyloidogenic mutations and finally develop and standardize bioinformatic diagnosis methodology incorporating clinical and LC-MS/MS data.

## Additional file

**Additional file 1.** Summary data for amyloidosis subtyping.

## Abbreviations

LCM: laser-capture microdissection
MS/MS: tandem mass spectrometry
HPLC: high performance liquid chromatography
SAP: serum amyloid protein
IHC: immunohistochemistry
SAA: serum amyloid A
AL: amyloid light-chain
ATTR: amyloid transthyretin
AA: amyloid type A
